# Photopharmacological Cholinergic Modulation of Cortical Activity in Human Brain Slices

**DOI:** 10.3390/brainsci16080822

**Published:** 2026-07-31

**Authors:** Jose Manuel Sanchez-Sanchez, Joana Covelo, Estefania Conde, Pedro Roldán, Jordi Rumià, Mar Carreño, Fabio Riefolo, Carlo Matera, Pau Gorostiza, Maria V. Sanchez-Vives

**Affiliations:** 1 Institute of Biomedical Research August Pi i Sunyer (IDIBAPS), 08036 Barcelona, Spain; jomasasa1@gmail.com (J.M.S.-S.); capela@recerca.clinic.cat (J.C.); 2 Unidad de Epilepsia, Hospital Clinic, 08036 Barcelona, Spain; econdeb@clinic.cat (E.C.); peroldan@clinic.cat (P.R.); jrumia@clinic.cat (J.R.); mcarreno@clinic.cat (M.C.); 3 Institute for Bioengineering of Catalonia (IBEC), 08028 Barcelona, Spain; friefolo@ibecbarcelona.eu (F.R.); cmatera@ibecbarcelona.eu (C.M.); pau@icrea.cat (P.G.); 4 Barcelona Institute of Science and Technology, 08028 Barcelona, Spain; 5 CIBER-BBN (Bioingeniería, Biomateriales y Nanomedicina), 28029 Madrid, Spain; 6 Catalan Institution for Research and Advanced Studies (ICREA), 08010 Barcelona, Spain

**Keywords:** photopharmacology, acetylcholine, slow oscillations, cortical network, neuromodulation, clinical neuroscience, human brain

## Abstract

**Highlights:**

**What are the main findings?**

- Photoactivation of Phthalimide-Azo-Iperoxo (PAI), a selective M_2_ muscarinic acetylcholine receptor agonist, can selectively modulate slow-oscillatory activity emerging from human cortical slices.
- PAI photoactivation induced a significant increase in oscillatory frequency and a corresponding reduction in both Up- and Down-state duration, confirming that the cholinergic system plays a critical role in regulating brain state transitions.

**What are the implications of the main findings?**

- To our knowledge, this is the first proof-of-concept study demonstrating the potential of photomodulation in human brain tissue, validating the feasibility of translating previous findings from animal models to human tissue.
- Selective light-mediated activation of muscarinic receptors in human brain tissue establishes a proof of concept for optical control of cholinergic signaling with high spatiotemporal precision.

**Abstract:**

**Background/Objectives:** Understanding the unique properties of human neurons is crucial for advancing knowledge of brain function and facilitating clinical translation. This study explores the use of photopharmacology, specifically a photoswitchable ligand, Phthalimide-Azo-Iperoxo (PAI), to modulate cortical activity in human brain slices. **Methods:** Human cortical tissue was obtained from patients undergoing resective neurosurgery for pharmacoresistant epilepsy. The inactive *cis* isomer of PAI (200 nM) was bath-applied to brain slices exhibiting slow-oscillatory activity, producing no effect on the ongoing network activity. Subsequent illumination of the slices with white light induced photoconversion to *trans*-PAI, the active form of the compound. **Results:** Photoactivation of PAI selectively activated M_2_ muscarinic acetylcholine receptors, resulting in a significant increase in oscillatory frequency accompanied by reductions in both Up-state and Down-state durations. **Conclusions:** In this proof-of-concept study, our findings provide preliminary evidence that photopharmacology can selectively modulate slow oscillations in human cortical circuits, highlighting its potential as a tool for investigating human cortical dynamics. By extending observations previously made in animal models to human tissue, this work establishes the feasibility of photopharmacological modulation in human cortical tissue and provides a foundation for future translational research.

## 1. Introduction

Understanding the unique properties of the human cerebral cortex is crucial for determining the mechanisms underlying brain function and pathology [1,2]. While most advances in basic neuroscience rely on animal models, research using human tissue samples and the development of advanced neuromodulation tools is highly relevant for potential future clinical applications [3]. Among these emerging technologies is photopharmacology, which combines optics and pharmacology to precisely target endogenous receptors in cell membranes [4]. Photoswitchable ligands are molecules whose structure can be altered with light, offering enhanced spatiotemporal control of activity without the need for genetic manipulation as in other light-controlled techniques like optogenetics [5,6].

In previous studies, we demonstrated that cortical brain activity can be modulated with light using a novel agonist of the M_2_ muscarinic acetylcholine receptor (mAChR), Phthalimide-Azo-Iperoxo (PAI), in animal models [7]. Given the cholinergic system’s central role in regulating numerous functions of the nervous system and its involvement in various neurological pathologies [8,9,10,11,12,13,14,15,16], the use of selective photoswitchable cholinergic drugs to modulate cortical activity holds significant scientific and clinical promise.

Brain slices from animal models have been widely used in studies investigating spontaneous slow oscillations (SOs)—a network rhythmic pattern of alternating active (Up) and inactive or silent (Down) states [17]. This activity generated by the cerebral cortex is considered the default emergent activity of the cortical network [18]. SO occur across various brain states, including physiological (slow-wave sleep), pharmacological (anesthesia), and pathological conditions (including stroke and traumatic brain injuries) [19,20]. While animal models provide substantial insights into cortical dynamics, human cortical slices have a special value since they provide the closest representation of healthy emergent activity in isolated human tissue [21], which comprises intrinsically distinct features from animal models [21,22,23,24]. Furthermore, the in vitro study of human cortex contributes to bridging the gap between basic neuroscience and clinical science, facilitating the translation of findings from animal models into human-relevant knowledge and improving the predictive value of preclinical research [24].

In the current study, we aimed to test the impact of photoactivation of M_2_ mAChR by PAI on spontaneous human cortical SO. To this end, we used human brain slices obtained from resective neurosurgery in patients suffering pharmacoresistant epilepsy. We demonstrated that light activation of PAI induces a selective control of SO in human brain slices, enhancing oscillatory frequency as a result of activation of M_2_ mAChR. Even though the number of observations is small, this study can be considered a proof of concept, and these results highlight the potential of photopharmacology to enhance our understanding of human-specific neural dynamics and lay the groundwork for the development of innovative therapeutic strategies.

## 2. Materials and Methods

### 2.1. Patient Information

Samples of human cortical tissue were obtained from Hospital Clínic de Barcelona from seven patients who underwent resective neurosurgery for refractory epilepsy. From these seven samples, we included four in this study, since the slices obtained from the remaining samples did not exhibit spontaneous SO. The information from the four patients (one female) included in the study is summarized in Table 1. Each of the *n* = 4 slices studied here were derived from a different patient. Surgery was performed at ages ranging from 27 to 48 years (mean ± SD = 37.50 ± 8.59 years). Three patients underwent anterior medial temporal resection, all of whom presented with hippocampal sclerosis, while one patient had a temporo-occipital lesion. Pre-surgical MRI findings included one case of focal cortical dysplasia type II, one patient with normal MRI results, one with an amygdala lesion, and one with cortico-subcortical gliotic changes. Anatomopathological analysis of the neocortex revealed that two patients presented apparently preserved neuronal architecture with minimal to no alterations, one patient exhibited a glioneuronal tumor suggestive of ganglioglioma and, in another patient, a polymorphous low-grade neuroepithelial tumor of the young (PLNTY) was detected in the analyzed sample. All patients experienced daily seizures, with epilepsy duration ranging from 8 to 28 years (mean ± SD = 21.00 ± 9.20 years).

### 2.2. Preparation of Human Cortical Slices

The resected tissue was transported to the laboratory in oxygenated cold (4–10 °C) bathing medium. Using a vibrating microtome (7000smz-2, Campden Instruments, Loughborough, UK), 400 μm thick coronal slices were cut from the samples provided by neurosurgery. To enhance tissue viability during slice preparation, we adapted the solutions used originally by Sanchez-Vives and McCormick [17] for ferret slices, with modifications to suit human tissue, including the addition of 3 mM sodium pyruvate and 0.5 mM ascorbic acid to the solution.

The slices were placed in an interface-style recording chamber (Scientific Systems Design Inc., Milton, ON, Canada) and bathed for 30 min in a mixture of the sucrose-substituted solution and artificial cerebrospinal fluid (ACSF). Following this, the slices were maintained in ACSF for an additional hour to allow for recovery, and then in a modified in vivo-like ACSF throughout the remainder of the experiment. The ACSF contained (in mM): NaCl, 126; KCl, 2.5; MgSO_4_, 2; NaH_2_PO_4_, 1; CaCl_2_, 2; NaHCO_3_, 26; dextrose, 10. The modified ACSF had the same ionic composition, with adjustments in KCl (4 mM), MgSO_4_ (1 mM), and CaCl_2_ (1 mM). All solutions were aerated with 95% O_2_ and 5% CO_2_ to maintain a final pH of 7.4, and the temperature was kept between 34.5 °C and 36 °C. Electrophysiological recordings commenced after a recovery period of at least 40 min following the transition to the modified ACSF.

### 2.3. Recording of Brain Activity and Data Analysis

**Figure 1:**
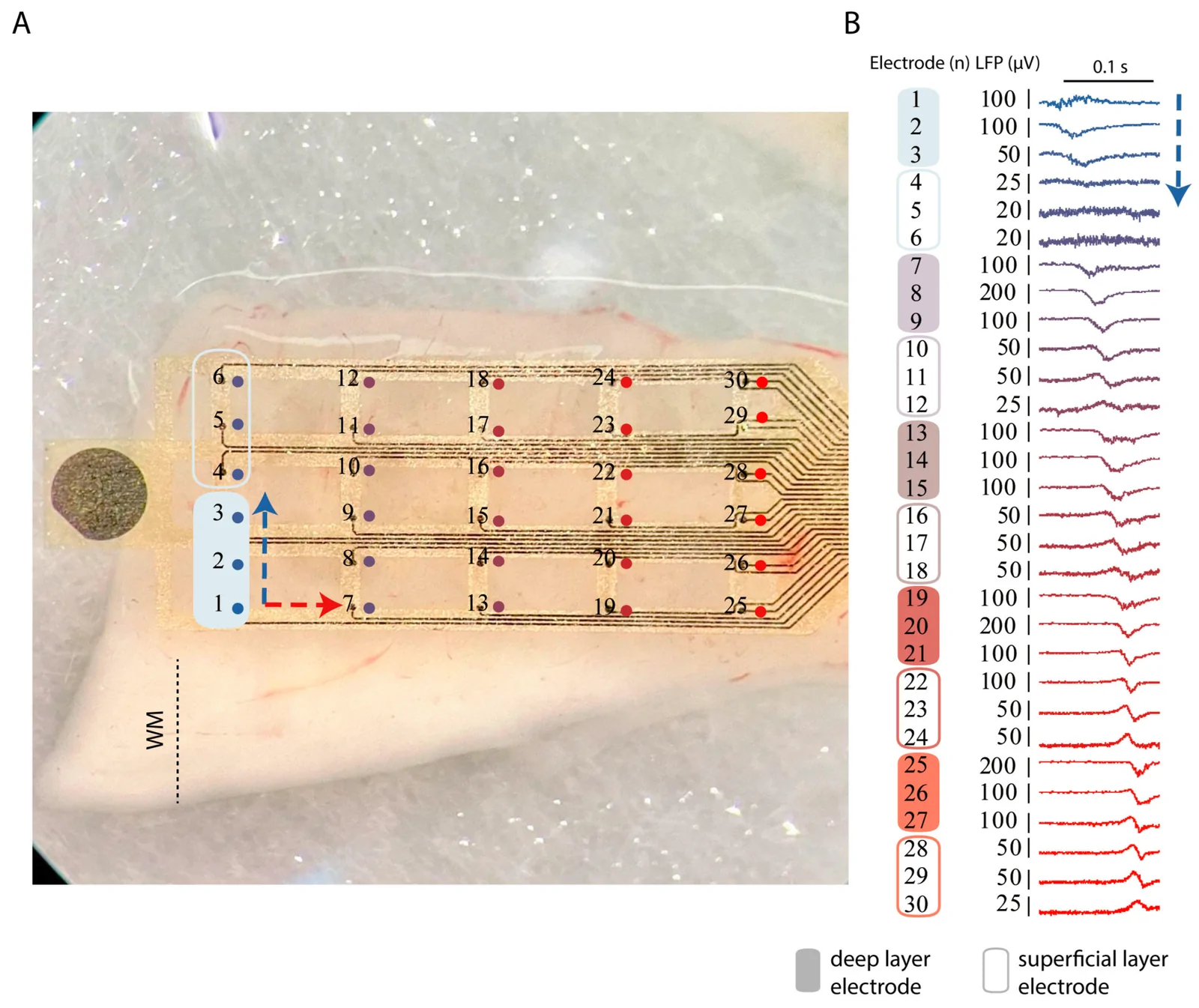
Human brain slice and local field potential. (**A**) Representative cortical slice obtained from resected human brain tissue and 30-electrode array used for data acquisition. (**B**) Representative local field potentials (µV) recorded during the control condition showing the propagation of a cortical Up-state from deep to superficial layers and from the left to the right part of the slice as a traveling wave. WM = White matter. The blue dashed arrow indicates deep-to-superficial propagation, whereas the red dashed arrow indicates left-to-right propagation. The colour code of channels (**A**) and recordings (**B**) is to facilitate channel identification and visualization.

Extracellular local field potentials (LFPs) were recorded using custom-made, flexible multielectrode arrays (MEAs) with 16 or 30 channels (CNM, Barcelona, Spain). The MEAs were positioned on the surface of the slice, spanning from the deep to superficial layers of the cortex (Figure 1A). The signal was amplified 100-fold using a PGA16 Multi Channel System Amplifier (MCS, Reutlingen, Germany), digitized with a Power 1401 CED (CED, Cambridge, UK) at a sampling frequency of 5 kHz, and acquired using the Spike2 v.9 software (CED, Cambridge, UK). We detected spontaneous spiking events at the population level corresponding to Up states and the subsequent silent Down states within the cortical network. To achieve this, the raw LFP signals (Figure 1B) were band-pass filtered between 200 Hz and 1500 Hz to estimate multiunit activity (MUA) [25]. The MUA reflects the firing activity of the local cortical network, and to account for large fluctuations in the signal, it was logarithmically scaled (logMUA). Event detection was then performed by applying a custom threshold to the logMUA signal, which was heuristically set following visual inspection [21]. This approach allowed us to determine the precise timing of Up-to-Down and Down-to-Up transitions for each event and recording channel. These transition times were used to calculate the duration of both Up and Down states, with the event durations represented at the population level using boxplots. Additionally, the average firing rate (FR) during each Up state was calculated as the mean value of logMUA throughout the Up-state duration.

### 2.4. Drug Application and Photostimulation in Human Brain Slices

PAI was synthesized as previously reported from commercially available materials [26]. PAI enables reversible photomodulation of M_2_ receptor activity both in vitro and in vivo. In its dark-adapted trans configuration, PAI acts as a potent M_2_ receptor agonist. Upon illumination with UV light (365 nm), it undergoes photoisomerization to the less active *cis* isomer. The active *trans* form can subsequently be restored by exposure to white light or by two-photon excitation using pulsed near-infrared light [7,26].

Accordingly, here we illuminated the PAI stock solution (100 µM) with UV light (365 nm) to induce the transition into *cis*-PAI, the less active isoform. Then, we bath-applied 200 nM of *cis*-PAI to the slices in the interface chamber. This application was performed in dark conditions to prevent photoconversion to *trans*-PAI. Following 1000 s, the slices were illuminated with direct white light to induce photoconversion from *cis*-PAI to *trans*-PAI (Figure 1A,B). The 200 nM concentration was determined to be optimal for photomodulation of cortical activity in brain slices due to the differences between the *cis* and *trans* isoforms of PAI [7]. In one out of the four slices used, the baseline activity was recorded following application and later washout of bicuculine methiodide (BMI, 8 μM), therefore possibly still containing BMI traces.

### 2.5. Statistical Analysis

Statistical analyses were performed in Python 3.8.5 using the *statsmodels* package (version 0.14.0). Linear mixed-effects models (LMMs) were fitted separately for oscillatory frequency, Up-state duration, Down-state duration, and firing rate. LMMs were used to account for the non-independence of repeated measurements obtained from the same cortical slice across experimental conditions. In each model, condition (Control, *cis*-PAI, and *trans*-PAI) was included as a fixed effect, while slice was included as a random intercept to account for repeated measurements obtained from each human cortical slice. Control was specified as the reference condition, and model parameters were estimated by maximum likelihood (ML; reml = False). Fixed-effect estimates (β), 95% confidence intervals (CIs), and Wald *z*-test *p*-values were reported for comparisons between treatment conditions and Baseline. Statistical significance was defined as *p* < 0.05.

## 3. Results

To investigate the local cortical spontaneous dynamics and its photopharmacological modulation in the human cerebral cortex, we used MEAs to record the spontaneous activity emerging from cortical slices in the absence of any chemical or electrical manipulation (Figure 1). We recorded LFP from *n* = 4 human cerebral cortex slices obtained from four different patients undergoing temporal resection due to refractory epilepsy (one from each patient). Although the tissue was obtained from patients with pharmacoresistant epilepsy, the slices were prepared from cortical tissue distant from the epileptic focus (as in [21]) and displayed spontaneous slow-oscillatory activity.

**Figure 2:**
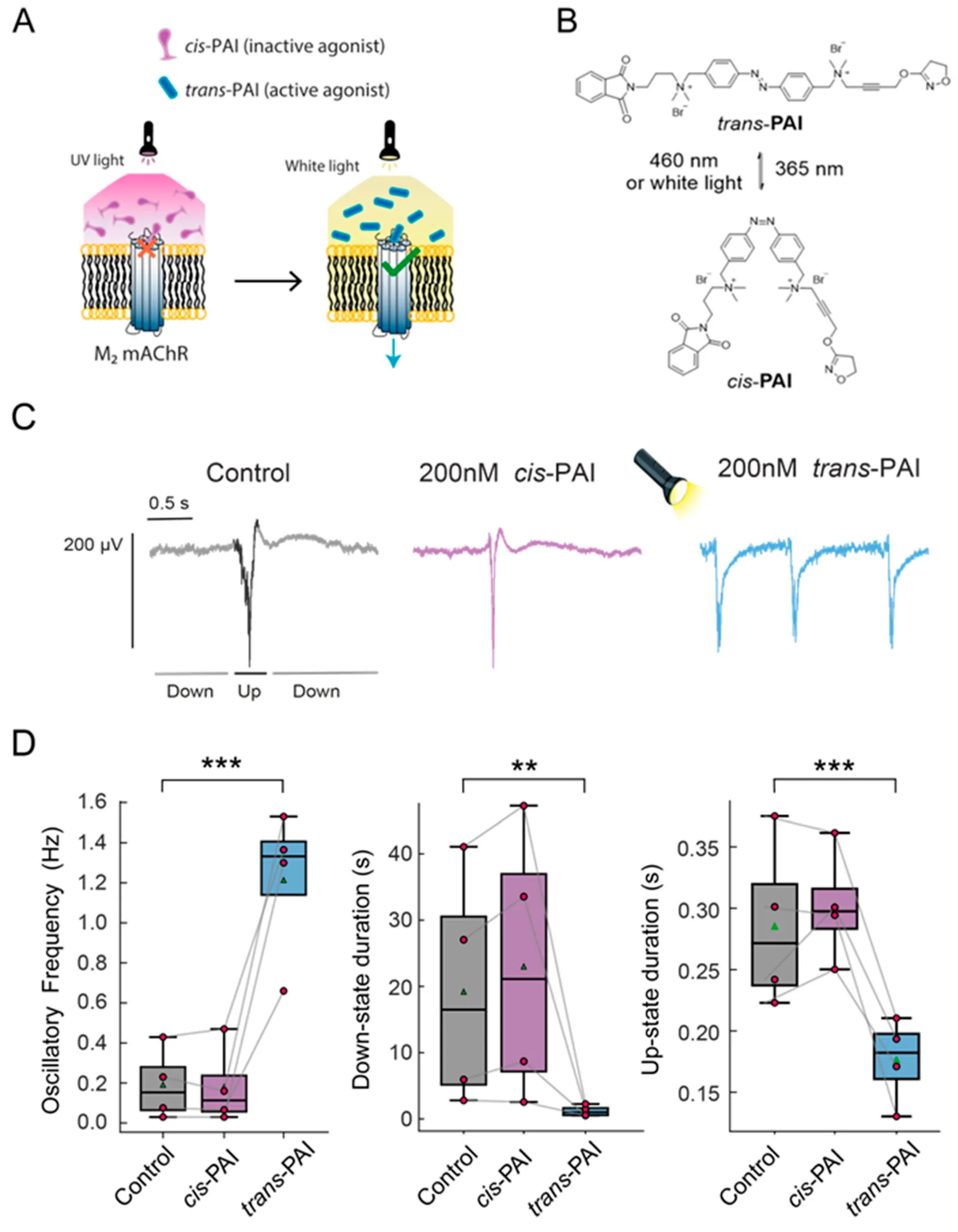
Photocontrol of slow oscillations in human brain slices using direct illumination of PAI with white light. (**A**) Schematic representation of the action of photocontrol of PAI on the M_2_ mAChR. The stock solution of PAI (100 µM) was pre-illuminated with UV light, resulting in *cis*-PAI, which is bath-applied to the brain slice at a concentration of 200 nM. Administration of this inactive agonist prevents receptor activation. Following a 1000 s incubation period, the tissue is illuminated with white light, prompting the photoconversion of cis-PAI to trans-PAI, and therefore enabling receptor activation. (**B**) Chemical structures of the *cis*- and *trans*- isomers of PAI, along with the wavelengths required for their photoswitching. (**C**) Representative traces of the local field potential (LFP) under control condition and following application of 200 nM pre-illuminated with 365 nm light (*cis*-PAI) and after photoconversion with direct white light, 200 nM PAI (*trans*-PAI). (**D**) Boxplot indicating the median value, black line, of oscillatory frequency (Hz, left), Down-state duration (s, middle) and Up-state duration (s, right) under the described conditions, with mean values represented by green triangles and individual values by red dots (*n* = 4). Differences between groups (control, *cis*-PAI and *trans*-PAI) are reported as *p*-values from a linear mixed-effects model with condition (Control, *cis*-PAI, *trans*-PAI) as a fixed effect and slice as a random intercept (** *p* < 0.01, *** *p* < 0.001, *n* = 4).

Our objective was to determine whether spontaneous network SO in human tissue could be modulated by activating a photoswitchable cholinergic M2 mAChR agonist, PAI. To do so, we recorded baseline SO as the control condition (Figure 1). Once we were able to record alternating Up and Down states, our preparation was considered suitable for the photopharmacology experimental protocol, as described in Barbero-Castillo et al. [7] (Figure 2A). SO consisted of Up states (periods of activity) interspersed with Down states (periods of silence) and displayed a frequency of 0.19 ± 0.18 Hz.

Subsequently, we bath-applied the inactive (*cis*) isoform of the photoswitchable M2 mAChR agonist, PAI. *Cis*-PAI was obtained by pre-illuminating the stock solution with 365 nm light under dark (Figure 2A). Following 1000 s, we illuminated the slice with white light to induce photoconversion of *cis*-PAI to *trans*-PAI, its active isoform (Figure 2B). Since this study was intended as a first proof-of-concept use of photopharmacological tools in human tissue rather than a dose–response or parameter-optimization study, which was previously investigated and reported [7], we have used the concentration of PAI that was considered to have the best *cis*/*trans* ratio in animal slices (200 nM) [7].

A linear mixed-effects model with condition (Control, *cis*-PAI, and *trans*-PAI) as a fixed effect and slice as a random intercept showed that *cis*-PAI did not significantly alter oscillatory frequency relative to Baseline (β = −0.010 Hz, 95% CI −0.251 to 0.231, *p* = 0.936, Figure 2C,D), whereas *trans*-PAI significantly increased oscillatory frequency (β = 1.022 Hz, 95% CI 0.781–1.263, *p* < 0.001, Figure 2C,D). The estimated between-slice variance was low (0.024 Hz^2^). Similarly, *trans*-PAI significantly reduced Up-state duration compared with Baseline (β = −0.109 s, 95% CI −0.151 to −0.067, *p* < 0.001, Figure 2C,D), while *cis*-PAI had no significant effect (β = 0.016 s, 95% CI −0.025 to 0.058, *p* = 0.442). For Down-state duration, *cis*-PAI did not differ significantly from Baseline (β = 3.794 s, 95% CI −9.322 to 16.910, *p* = 0.571, Figure 2C,D), whereas trans-PAI was associated with a significant reduction (β = −18.017 s, 95% CI −31.133 to −4.901, *p* = 0.007, Figure 2C,D). The estimated between-slice variance was 102.7 s^2^, reflecting substantial variability among slices. Neither *cis*-PAI nor *trans*-PAI significantly affected firing rate relative to Baseline. *Cis*-PAI produced a non-significant change in firing rate (β = −0.078 a.u., 95% CI −0.281 to 0.126, *p* = 0.454), and *trans*-PAI likewise had no significant effect (β = 0.009 a.u., 95% CI −0.194 to 0.212, *p* = 0.929). The estimated between-slice variance was 0.080 a.u. ^2^, indicating modest variability across slices.

**Figure 3:**
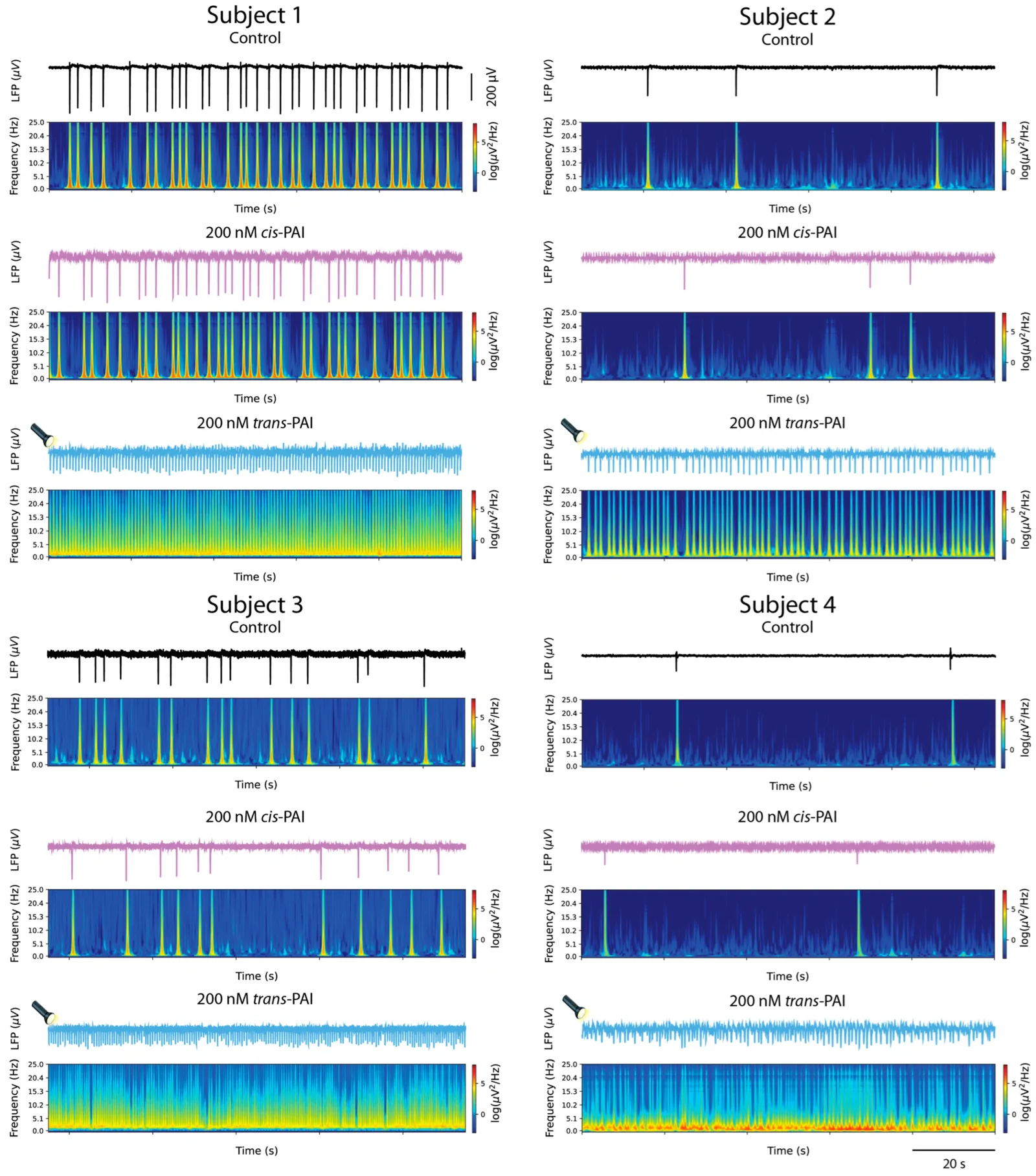
Photoactivation of M_2_ mAChR associated with changes in the slow-oscillatory regime. Representative local field potential (LFP) traces and spectrograms of the activity recorded from each subject (one slice per subject) during control (**top**), application of inactive isoform of PAI (*cis*-PAI, **middle**) and after photoactivation of M_2_ mAChRs due to direct illumination of slices with white light (photoconversion to *trans*-PAI, **bottom**).

Overall, the linear mixed-effects model identified significant effects of *trans*-PAI on oscillatory frequency and Up- and Down-state duration, but not in firing rate. The average values for these parameters, as well as their standard deviation and *p*-values, are presented in Table 2. The modulation of PAI was clear in all slices included in this study (*n* = 4), each slice deriving from a different patient, as can be observed in Figure 3, even when departing from distinct oscillatory levels.

## 4. Discussion

In this proof-of-concept study, we demonstrate the feasibility of photopharmacology for modulating neural activity in human cortical slices, specifically by targeting M_2_ mAChR using the photoswitchable agonist PAI. Our results confirm that spontaneous SO, a prominent cortical emergent pattern under physiological and pathological conditions, can be selectively controlled in human brain tissue using light-controlled modulation. A previous study has demonstrated the effectiveness of PAI in modulating cortical activity in animal models [7]; however, to our knowledge, the current study is the first to validate and extend these findings and use photopharmacology in human brain tissue. A large part of existing reports in human cortical slices have relied on proconvulsant agents or modifications to the ACSF to enhance the levels of excitability of the network, mostly for the study of epilepsy [27,28,29]. However, spontaneous activity under physiological conditions, as shown in this study (Figure 1), is less frequently reported and remains poorly understood [21,29]. Since the tissue used in this study was obtained from the brain of patients with refractory epilepsy, the recorded activity might display signs of hyperexcitability [21], despite the fact that the slices were cut from cortical blocks located distant from the epileptic focus. Since, for ethical reasons, neither fresh brain tissue nor intracranial recordings can be obtained from healthy individuals, the activity described here represents, in any case, the closest approximation to healthy activity from isolated human cortex [21].

Here, we demonstrate that the spontaneous slow-wave activity emerging from human cortical slices can be modulated using PAI, a photoswitchable muscarinic M_2_ mAChR agonist. PAI activation with light can induce a significant increase in oscillatory frequency and a corresponding reduction in both Up- and Down-state, when comparing both with the baseline and *cis*-PAI (inactive) conditions, (Figure 2 and Figure 3). Such an enhancement in excitability is similar to the one during the transition from slow-wave to REM sleep or to awakening, as well as during the awakening from anesthesia [30,31,32], state transitions in which the ascending cholinergic system plays an important role. Similarly, in a previous study using an animal model, M2 mAChR photoactivation resulted in an increase in oscillatory frequency and a decrease in firing rate during the Up state [7]. Here, we also observed a significant increase in oscillatory frequency, but the firing rate did not significantly vary. Moreover, while the decrease in Down-state duration was observed in both studies, here we observed a significant decrease in Up-state duration, which was not reported in the animal model. These differences could result from the specific response of human tissue to M2 activation. Species differences in the laminar and cellular distribution of M2 mAChR, together with differences in cortical microcircuit organization, may contribute to the distinct responses observed across species, although the overall role of M2 mAChR appears to be broadly conserved across mammals [33].

The findings reported here in human brain slices build on previous studies in animal models by demonstrating the feasibility of photopharmacological modulation in human tissue, suggesting new opportunities for the selective modulation of neural circuits associated with cholinergic dysfunction [34]. By enabling light-controlled activation of specific receptor populations, photopharmacology may provide a valuable experimental platform for investigating circuit mechanisms and could inform the development of future therapeutic strategies for conditions such as Alzheimer’s disease and schizophrenia [11,35].

However, substantial work is required before clinical translation can be considered. Future studies should validate these findings in larger cohorts, characterize the long-term effects and safety of M_2_ mAChR photomodulation, establish the pharmacokinetic and toxicological profile of PAI, and further develop high-resolution light-delivery methods suitable for selective activation in the brain. It is important to acknowledge the limitations of this study, particularly the small sample size. The data were obtained from cortical slices derived from only four patients, which limits the generalizability of our findings. However, the study provides proof of concept that light-controlled pharmacological modulation of muscarinic receptors can be achieved in human cortical tissue. The variability inherent in human tissue, especially from individuals with pharmacoresistant epilepsy, may introduce additional complexities that require careful consideration.

In conclusion, our findings provide the first demonstration of photopharmacology as a tool to investigate the dynamics of the human cortex and the role of M2 mAChRs in cortical activity. By extending previous observations from animal models to human brain tissue, this proof-of-concept study establishes a foundation for future investigations of photopharmacological approaches in human cortical circuits. Further studies with larger sample sizes will be necessary to assess the robustness, generalizability, and potential translational relevance of these findings.

## Figures and Tables

**Table 1:** Demographic, Clinical, and Neuropathological Characteristics of Patients.

ID	Sex	Age	Dur (y)	Surgery	Diagnosis	MRI Features	Neocortex Pathology	Seizures	Engel	HS
**1**	M	38	8	Left anterior medial temporal resection	Epilepsy	Focal cortical dysplasia type II	Preserved neuronal architecture with patchy neuronal pyknosis	Daily	III	Yes
**2**	M	37	27	Right anterior medial temporal resection	Epilepsy	No alterations	No alterations	Daily	I	Yes
**3**	F	27	21	Right anterior medial temporal resection	Epilepsy	Right amygdala lesion associated with mesial sclerosis	Glioneuronal tumor suggestive of ganglioglioma	Daily	I	Yes
**4**	M	48	28	Right neocortical temporo-occipital resection	Epilepsy	Cerebral cortex globally preserved with cortical–subcortical gliotic changes	Polymorphous low-grade neuroepithelial tumor of the young (PLNTY)	Daily	I	No

Abbreviations: Dur (y), Duration of epilepsy in years; Sex: M (Male), F (Female); HS, Hippocampal Sclerosis; Engel, Engel Surgical Outcome Scale (Class I: Free of disabling seizures; Class III: Worthwhile improvement).

**Table 2:** Oscillatory parameters during control condition and photoconversion of PAI isoforms. Differences between conditions (control, *cis*-PAI and *trans*-PAI) are reported as *p*-values from a linear mixed-effects model with condition as a fixed effect and slice as a random intercept (** *p* < 0.01, *** *p* < 0.001 *n* = 4).

Parameter	Control (Mean ± SD)	*cis*-PAI (Mean ± SD)	*trans*-PAI (Mean ± SD)	*p*-Value *n* = 4 (Control-*cis*-PAI)	*p*-Value *n* = 4 (Control-*trans*-PAI)
Oscillatory frequency (Hz)	0.19 ± 0.18	0.18 ± 0.20	1.21 ± 0.38	0.936	<0.001 ***
Up-state duration (s)	0.29 ± 0.07	0.30 ± 0.05	0.18 ± 0.03	0.442	<0.001 ***
Down-state duration (s)	19.22 ± 18.11	23.02 ± 20.99	1.21 ± 0.82	0.567	0.007 **
Firing rate Up (a.u)	0.80 ± 0.45	0.73 ± 0.28	0.81 ± 0.36	0.454	0.929

## Data Availability

The data supporting the findings of this study are openly available in Zenodo at https://zenodo.org/records/20750122 (accessed on 29 July 2026).
